# Cultivated Land Transfer, Management Scale, and Cultivated Land Green Utilization Efficiency in China: Based on Intermediary and Threshold Models

**DOI:** 10.3390/ijerph191912786

**Published:** 2022-10-06

**Authors:** Min Zhou, Hua Zhang, Nan Ke

**Affiliations:** 1 School of Public Management, Liaoning University, Shenyang 110036, China; 2 Sunwah International Business School, Liaoning University, Shenyang 110036, China; 3 College of Public Administration, Huazhong University of Science and Technology, Wuhan 430079, China

**Keywords:** cultivated land transfer, cultivated land green utilization efficiency, cultivated land management scale

## Abstract

Cultivated land utilization around the world is accompanied by the cultivated land fragmentation, which is a significant agricultural feature of countries with economies in transition. Thereby, governments of the PRC have successively promulgated a series of relevant policies to promote the cultivated land transfer (CLT) and stimulate the transformation of cultivated land utilization to be both green and efficient. In the context of large-scale CLT and the implementation of a rural revitalization strategy for China, it is of great significance to explore the effect of CLT on cultivated land green utilization efficiency (CLGUE). In this work, 30 provinces of China were selected as the objects of investigation; the super-efficiency SBM model was used to evaluate CLGUE; the mediation effect model and threshold regression model were used to gain a more comprehensive understanding of the CLT’s influence on CLGUE. According to the results of this study, the following conclusions were drawn. First of all, the CLGUE in China as a whole showed an upward trend improvement from 2005 to 2019. Due to the different natural and economic conditions, the CLGUE trends showed significant spatial disparities at both the grain functional areas level and provincial level. Secondly, the CLT could promote CLGUE directly, and the mediation regression results demonstrated that CLT was able to enhance CLGUE indirectly through the mediator of cultivated land management scale. Thirdly, the threshold effect test confirmed the existence of a single threshold, indicating that when the level of CLT gradually crossed the threshold, the promotion effects of CLT on CLGUE would slow down. Lastly, the heterogeneity analysis indicated that the promotion effects of CLT on CLGUE in different geographical location areas and grain functional areas were positive, and that there were significant differences in regression coefficients.

## 1. Introduction

**Figure 1:**
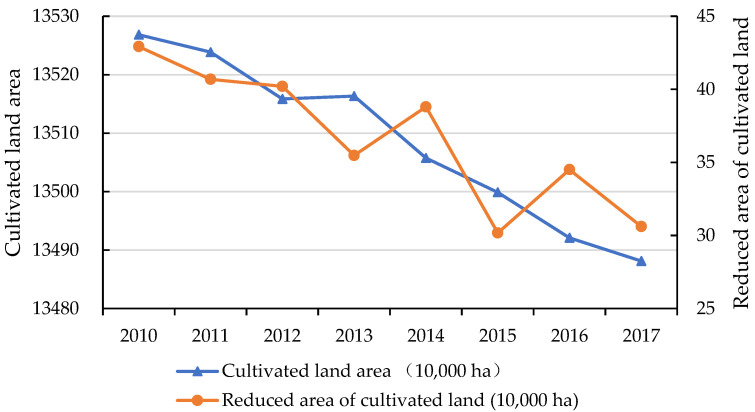
Cultivated land area and cultivated land area reduced in China.

According to the UN statistics, more than 1.86 billion people will live in urban areas from 2009 to 2050, and the urbanization rate is estimated to increase from 50 to 69% [1]. In China, the urbanization rate, reflected by urban population, increased from 10.64% in 1949 to 63.89% in 2020. Urbanization is an important way of promoting and effective carrier of agricultural surplus labor transfer in the process of economic transition [2,3]. However, considering the basic national conditions in China with a large population and little land, the contradiction of the expansions of urban construction and cultivated land protection is becoming prominent. The large-scale spatial expansion of urbanization has brought the reduction of cultivated land and food security. The rapid process of urbanization has caused a huge quantity of cultivated land to be lost [4], especially after 2010 (Figure 1). According to the “China Land and Resources Statistical Yearbook (2017)”, the area of cultivated land in China had reduced to 134,863,200 hectares (2.023 billion mu). The shortage of cultivated land has become more and more serious, which is threatening the food security and social stability. In addition, with the transfer of young and middle-aged rural labor from agricultural to non-agricultural sectors during the process of urbanization, the problem of who will farm the cultivated land in future has become another focus of urgent attention in China.

The Chinese government has successively promulgated a number of related laws and regulations, such as improving the eco-friendly utilization of cultivated land, implementing the policy of agricultural subsidies, encouraging the practice of cultivated land transfer (CLT), to alleviate the shortage of cultivated land and solve the problem of who farm cultivated land. Based on “Statistics from China Rural Statistical Yearbook (2021)”, grain yield of China had grown from 318.7 kg per capita in 1978 to 474.4 kg per capita in 2020. However, high yields may be the results of unsustainable farming methods [5]. For example, the use of cultivated land has contributed to massive carbon emissions growth in China. The carbon emissions from agricultural land utilization in mainland China is on the rise, from 5232.83 thousand tons in 2000 to 7613.31 thousand tons in 2017 [6]. Moreover, the fertilizer amount applied per unit of cultivated land is much higher than the world average level and the maximum limit, and the as the applied amount of chemical fertilizer is 1.6 times of the world average [7]. Excessive use of fertilizers has become the main source of agricultural non-point source pollution in China [8], which seriously threatens soil safety [9].

More attention should be paid to the ecological and environmental effects of the process of cultivated land utilization. Since Schaltegger and Sturm (1990) [10] proposed the conception of ecological efficiency in the 1990s, the core of eco-efficiency lies in the introduction of economic and ecological dimensions into the terms of production evaluation [11]. The concept of eco-efficiency has been applied to solve problems in various fields, especially in industrial research [12] and regional research [13]. As for the eco-efficiency of cultivated land use, the relevant literature covers the following aspects: (1) The design and method of evaluation. The existing research usually measures the ecological efficiency of cultivated land use comprehensively, and the evaluation indexes were selected from “input”, “desirable output”, and “undesirable outputs” [14]. The carbon emission and agricultural pollution were included in the evaluation of ecological efficiency of cultivated land use as the undesired output [15,16,17]. In terms of evaluation method of the cultivated land utilization eco-efficiency, super-efficient SBM model [14,16], comprehensive index evaluation [18], SFA model [19], three-stage super-efficiency SBM-U model [20], and non-radial directional distance function (NDDF) approach [21] were used. (2) The spatial-temporal evolution of cultivated land utilization eco-efficiency. Ma et al. (2022) [17] explored the characteristics of spatial–temporal changes of ecological efficiency of cultivated land use in thirty-one provinces in China from 2000 to 2019. The result indicated that the ecological efficiency of cultivated land use in all provinces reduced from 0.408–3.976 to 0.353–2.046. However, Ke et al. (2022) [14] reported a rise in eco-efficiency of cultivated land use from 0.4393 to 0.8929 from 2000 to 2019 in China. (3) The determining issues of cultivated land utilization eco-efficiency. Empirical studies revealed that the eco-efficiency of cultivated land use was affected by resource endowments, economic development level, natural conditions, production conditions [17], household livelihoods, adjustment of planting structure, the stability of land property rights and the household cultivated land scale [22]. Taking the Yangtze River Economic Belt in China as the research target, Hou et al. (2019) [16] discussed the relationship between urbanization and ecological efficiency of cultivated land use. The research concluded that urbanization affects the cultivated land utilization eco-efficiency by affecting the combination of input and output. 

At present, in the general trend of eco-friendly and sustainable utilization of resources, China has been supporting the transformation of cultivated land use to become green and efficient [23]. However, the concept and meaning of the cultivated land green utilization efficiency (CLGUE) are still unclear [24]. Xie et al. (2018) interpreted CLGUE as combining the largest economic and ecological effects that are generated with the lowest cost from cultivated land use [21]. In other words, CLGUE is not only involves the economic outputs, but also takes the positive and negative externalities during cultivated land utilization into account. Ke et al. (2021) integrated the concept of “green” and “low-carbon” involved into the measurement of CLGUE [25].

Centralizing cultivated land from aged farm households to young professional investors, and implementation of farmland professional, technical and moderate-scale management are effective paths to solve the problem of who farm cultivated land in the future China. In 2014, the central governments of CPC issued “Opinions on Guiding the Orderly Circulation of Rural Land Management Rights to Develop Moderate Scale Operation of Agriculture”, which required that CLT and moderate-scale of cultivated land management should be energetically developed. In addition, the China’s government promulgated the “Three Rights Separation Policy” to further separate the right to manage cultivated land from the right to contract the management of cultivated land. Ownership, contract right and management right are separated from each other. With the progress of rural reform, the action supporting cultivated land as an advantage has been unceasingly strengthened, and the CLT has developed rapidly [8]. As evidence thereof, the area of contracted land has exceeded 104 million hectares, and the area of cultivated land with transferred use rights was 35.48 million hectares, accounting for more than one third of the total contracted land, in 2020. 

In recent years, scholars have shown mounting interest in the performance of CLT, and most of the current research mainly involves the following aspects: (1) The current literature focuses more on the influence of CLT on the agricultural environment [26], income of rural households [27], households’ behaviors for cultivated land utilization [28], pollution from non-point agricultural source [29], fertilizer utilization and PM 2.5 pollutants [30]. (2) The investigation methods, including the mediating effect model [26], agent-based model (ABM), substance flow analysis (SFA) [31], endogenous switching model [32] and Granger causality test [30] were used. In addition, there is also some of the literature that explored the impact of CLT on cultivated land utilization. Taking Daligang Township (located in the typical subtropical hilly area of China) as a case study area, Yuan et al. (2016) performed an empirical study on the synergistic effects of CLT on rice planting and nitrogen utilization. The results found that along with CLT, the circulated cultivated land was gradually taken over by balanced households, farming-oriented households or large-scale households. They increased the double-rice planting rates and rice yields [31]. The study of Gu et al. (2017) found that an increase in plot size implies a decrease in the cost of machine utilization, an increase in the possibility of mechanical utilization and cultivated land investment, and a decrease in the labor input per unit area [33]. After collecting the information of 30 provinces in China from 2000 to 2017, Fei et al. (2021) constructed a counterfactual framework using PSM method to analyze the impact of CLT on cultivated land utilization efficiency. The analysis showed that the cultivated land utilization efficiency in the provinces with CLT was higher than that in the provinces without CLT [34]. According to the information of 892 farmers from ten counties in Shandong, China, Zheng et al. (2021) estimated the impact of different CLT characteristics on farmers’ fertilizer application. The results showed that farmer’s chemical fertilizer input decreased significantly after CLT introduction, while increased significantly after CLT removal, and CLT mainly affected farmers’ fertilizer application by controlling the supply of agricultural labor [32]. The empirical research of Guo et al. (2022) also showed that CLT could reduce the quantity of fertilizer used, and the influence of CLT on PM 2.5 pollution was negative [30].

With the acceleration of urbanization, CLT has become an increasingly common strategy to increase the circulated land utilization efficiency and mitigate the fragmentation of small farmland [31]. The change of cultivated land management scale (CLMS) brought by CLT will change the allocation of resources, and how this change will affect CLGUE is rarely paid attention by scholars. In conclusion, the existing literature has paved the way for the development of our work, but at least the following two points need to be further improved. Firstly, the research on the influence and mechanism of CLT on CLGUE needs to be strengthened. Secondly, the existing literature usually ignored the relationship between CLT and CLGUE, one-sided analysis of a single link not being conducive to an accurate understanding of the effect of CLT on CLGUE. The influence of CLT on CLGUE is an interlocking process; therefore, the direct estimation of the impact of CLT on CLGUE may lead to errors in the estimation results.

In view of China’s large-scale CLT and rural vitalization strategy, it is significant to explore the effect of CLT on CLGUE and its underlying mechanisms. The possible contributions to the literature of this work were mainly made in three points. Firstly, considering the influence of CLT on CLGUE is an interlocking process, this paper uses the mediation effect model to measure the impact of CLT on CLGUE, which can effectively solve the errors of the direct estimation and can be deemed as an improvement over previous research. Secondly, compared with the traditional method of regional division, the classified method of food functional areas is used in this paper, which has advantages in objectively reflecting the level of regional cultivated land resource utilization, and food production and distribution. Lastly, this paper conducted an in-depth analysis on the influence of CLT on CLGUE, so as to provide a basis for targeted policy formulation on the improvement of CLGUE. The main contents of this work are as follows. Firstly, thirty provinces in Chinese Mainland are taken as the research objects of this work, and the mediation effect model is used for an overall view of the CLT’s impact on CLGUE. Secondly, a threshold regression model is used to test the threshold effect between CLT and CLGUE. Lastly, this study considers the regional differences in CLT on CLGUE from the perspectives of both geographical location and grain functional areas. 

## 2. Analytical Framework and Research Hypotheses

### 2.1. Cultivated Land Transfer and Cultivated Land Green Utilization Efficiency

The influence path of CLT on CLGUE mainly includes the following aspects. Firstly, the level of cultivated land fragmentation can be reduced by concentrating cultivated land. Under the background of the national resource situations of large population and little-cultivated land, the assignment of cultivated land in accordance with the Household Responsibility System (HRS) according to quality and location makes China’s cultivated land fragmented [35]. Due to the increasing area of ridges and ditches, fragmentation of cultivated land wastes cultivated land resources and agricultural operation time, reduces irrigation efficiency, and causes inconvenience in field management [36]. In addition, there are some studies demonstrating that the fragmented cultivated land is crucial for losing agricultural production technical efficiency [37]. CLT enables farmers to adjust the scale of cultivated land management and reduce the fragmentation of cultivated land [38].

Secondly, the efficiency of the allocation of cultivated land resources can be increased by CLT. According to neoclassical theory, homogeneous elements need to be rewarded equally in the perfectly competitive markets. This stands for the marginal output of factors being equal for all producers regardless of their production efficiency. However, factors will be transferred to the producers with higher marginal output. On the one hand, CLT makes it possible for farmers with high efficiency to expand the scale of cultivated land management. On the other hand, due to the low marginal output of cultivated land, farmers with low efficiency can reduce their scale of cultivated land management by transferring out their cultivated land. Therefore, the transformation of cultivated land from inefficient farmers to efficient farmers can increase the allocation efficiency of cultivated land resources [39]. In addition, CLT promotes the transformation of agricultural management from part-time and small-scale farmers to specialized and large-scale farmers, improving agricultural management capacity and agricultural planting technology while extending the scope of cultivated land management [40].

Lastly, the practices of cultivated land utilization are being made more eco-friendly. CLT transfers the cultivated land from part-time and small-scale farmers to large growers, family farms, cooperatives and other specialized cultivation direction concentration. The operators who have higher operational capacity and rich production experience are more likely to adopt advanced agricultural machinery and eco-friendly and environmental planting technology. Based on the questionnaire survey of 191 respondents in four counties of Jiangsu Province, Zhu et al. (2017) explored the influence of the cultivated land size in circulation on fertilizer input, and the result showed that farmland’s scale has an obvious negative effect on fertilizer input [41]. In addition, based on the agricultural production data covering 26 provinces from 2007 to 2016, Ma et al. (2019) studied the effect of CLT on agricultural environmental efficiency. They found that CLT shows a positive and direct impact on agricultural environmental efficiency under the “non-point source pollution” and “carbon emissions”. CLT can effectively improve production efficiency with more rational resource allocation and make up for the shortcomings in the application of green technologies so as to promote the ecological and effectual use of cultivated land [26]. Therefore, this study put forward the following assumption:

**Hypothesis** **1** **(H1).** *CLT shows a substantial positive effect on CLGUE.*

### 2.2. Cultivated Land Transfer and Cultivated Land Management Scale

As urbanization and industrialization accelerate, many rural laborers are transferred to urban areas, leading to the growth of CLT. For the past decade, more and more farmers are engaged in CLT to expand CLMS in China. According to the data from the “China Rural Management Statistical Annual Report” from 2005 to 2020, the area of transferred farmland increased from 3.64 million hectares to 35.48 million hectares, with a substantial increase of the proportion from 4.57% to 36.16%. CLT is essential for management in scale of cultivated land [42]. CLT scales up the operation of cultivated land. In the future, the proportion of large-scale households in agricultural production will continuously grow and become larger [43]. According to the data of 26 provinces in China from 2007 to 2016, Ma et al. (2019) evaluated the influence of CLT on CLMS, which suggested that CLT helped to extend CLMS (size per agricultural labor) [26]. Therefore, this study proposes the following assumption:

**Hypothesis** **2** **(H2).** *CLT has a positive effect on CLMS.*

### 2.3. Cultivated Land Management Scale and Cultivated Land Green Utilization Efficiency

The influence path of CLMS on CLGUE mainly includes the following aspects. Firstly, the total production cost per unit area can be reduced by the amplification of CLMS. Due to the inseparability of agricultural infrastructure and other production issues, the miniature cultivated land management restricts the efficiency of resource utilization and has input redundancy. The expansion of management scale has a significant impact on the optimal allocation of resources [44]. According to the survey data of 331 rice growers in northeastern Jiangxi Province, China, Tan et al. (2008) reported that the increase in farm size decreases the total production costs per ton, and the production cost per unit yield would be reduced by 1.4% if the CLMS increased by 1 mu [45]. On the basis of the field survey data of 1049 farmers in 100 villages in 5 provinces, China, Xu et al. (2011) found that, except for japonica rice, the development of cultivated land management had a considerable negative effect on the total production cost per unit yield [46]. According to the micro-survey data on farmers from eight major grain-producing provinces from 2003 to 2013 at fixed observation points in rural areas of the Ministry of Agriculture of China, the research of Tang et al. (2017) found that the development of farmers’ CLMS significantly reduced the production cost per unit mu [47]. 

Secondly, the carbon and pollution emissions from cultivated land use can be lowered though agricultural chemical reduction. During the rapid development of urbanization, the continuous transfer of rural laborers to non-agricultural industries leads to the relative shortage and rising price of labor. However, the amplification of CLMS further aggravates the shortage of labor after large-scale CLT. Under the condition of inelastic labor supply, the labor-saving technology (labors were substituted by agricultural machinery) has been a realistic choice for agricultural development and micro-farmers [48]. Studies have shown that mechanized operations such as mechanical land preparation, deep tillage and mechanical fertilization had a considerable effect on cultivated land quality and fertilizer utilization efficiency [49]. Furthermore, the estimation results of Zhu et al. (2017) showed that farmland’s scale has an obvious negative influence on fertilizer input [41]. 

Lastly, the cultivated land productivity can be increased by the deepening division of labor and specialization. The amplification of CLMS leads to the deepening of the division of labor and specialization [44], which has a significant on improving cultivated land productivity. Mugera and Langemeier (2011) examined the technical and scale efficiency scores for a balanced panel of 564 farms in Kansas, USA, from 1993 to 2007. The empirical results showed that the larger farms were industrially more efficient than the smaller ones [50]. On the basis of household surveys of the core region of food production in Henan Province, China, Liang et al (2016) explored the influencing issues of household cultivated land utilization efficiency which considering the environmental factors. The investigation reflected that the development of household cultivated land provides a positive effect for cultivated land utilization efficiency, but actually, small-scale cultivated land and fragmentation land are widespread, which reduces the positive impact they have [22]. 

Additionally, the CLMS in most provinces and regions of China has not reached the optimal level [51]. In view of the limited CLMS brought by the basic national situations of large population and little cultivated land, the allocation of cultivated land under HRS based on quality and location will inevitably cause a series of fragmented cultivated land plots [38]. As evidence thereof, each rural household manages a small piece of cultivated land allocated from the rural collective to which it belongs, averaging 0.5 hectares per household under HRS [52]. The ultra-small-scale operation of cultivated land especially occurs in the grain production core area, the research in Henan province has shown that the average number of plots per household is 4.13, and the average area of each cultivated land is lower than 0.1 hectares [22]. Compared with the requirements of agricultural modernization, these small plots seriously impede the widespread application of large farm machinery [53], and the excessively scattered agricultural ultra-small-scale management has shown remarkable inadaptability in China. CLT is the most important means to promote appropriate management scales of cultivated land in China [54]. In conclusion, the present study put forward the following assumptions:

**Hypotheses** **3** **(H3).** *CLMS is positively related to CLGUE.*

**Hypotheses** **4** **(H4).** *CLT can enhance CLGUE through the mediator of CLMS.*

**Figure 2:**
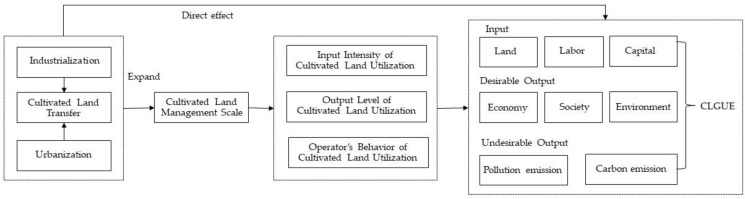
Analysis framework.

The overall analytical framework is shown in Figure 2.

## 3. Materials and Methods 

### 3.1. Model Construction

#### 3.1.1. CLGUE Evaluation Model

Charnes et al. (1978) initially proposed the data envelopment analysis (DEA) model [55]. The DEA model is a holistic factor analysis model with multiple indicators, and it is favored because it can objectively determine the weight of the parameters and evaluate the efficiency of the system [21]. Efficiency measurement with the traditional DEA model is primarily based on the radial and angle levels of homogeneous units to minimize the input or maximize the output. However, it largely ignores the undesirable output (i.e., inefficiency) in the evaluation process [15]. Tone developed a non-radial and non-angular slack-based measure (SBM) model to improve this problem [56]. The super-efficiency SBM model has the advantages of both the SBM model and the super-efficiency of DEA model, which can measure efficiency incorporating undesirable outputs, and also distinguish and compare the effective decision-making units so as to avoid the loss of information of effective decision-making units [25,57]. 

Therefore, we used the super-efficient SBM model to evaluate the CLGUE. The following formula describes the principle of the model with unexpected output. The number of decision-making units (DMUs) of the cultivated land use is n. m is the number of input types; $S_{1}$ and $S_{2}$ are the number of desirable and undesirable output types, respectively. $x\in R^{m},y^{g}\in R^{s_{1}},y^{b}\in R^{s_{2}}$ correspond to the input, desirable output and undesirable output, respectively. The matrix can be described as: $X=\left[x_{1},\cdots ,x_{n}\right]\in R^{m\times n}\;$, $Y^{\;g}=\left[y_{1}^{g},\cdots ,y_{n}^{g}\right]\in R^{s_{1}\times n}$, $Y^{\;b}=\left[y_{1}^{b},\cdots ,y_{n}^{b}\right]\in R^{s_{2}\times n}$. The super-efficiency SBM model containing the unexpected output can be detailed by following formula [25]:

(1) $$\rho^{*}=min\frac{1+\frac{1}{m}\sum_{i=1}^{m}\frac{D_{i}^{-}}{x_{ih}}}{1-\frac{1}{S_{1}+S_{2}}\left(\sum_{r=1}^{S_{1}}\frac{D_{r}^{g}}{y_{rh}^{g}}+\sum_{k=1}^{S_{2}}\frac{D_{k}^{b}}{y_{kh}^{b}}\right)}$$

(2) $$s.t.\{\begin{array}{c} x_{ik}\geq \overset{n}{\underset{j=1,\;j\neq h}{\sum }}\lambda_{j}x_{ij}-D_{i}^{-},i=1,\cdots ,m\\ y_{rh}^{g}\geq \overset{n}{\underset{j=1,\;j\neq h}{\sum }}\lambda_{j}y_{rj}^{g}+D_{r}^{g},r=1,\cdots ,s_{1}\\ y_{kh}^{b}\geq \overset{n}{\underset{j=1,\;j\neq h}{\sum }}\lambda_{j}y_{kj}^{b}-D_{r}^{b},k=1,\cdots ,s_{2}\\ 1-\frac{1}{s_{1}+s_{2}}\left(\overset{s_{1}}{\underset{r=1}{\sum }}\frac{D_{r}^{g}}{y_{rh}^{g}}+\overset{s_{2}}{\underset{k=1}{\sum }}\frac{D_{k}^{b}}{y_{kh}^{b}}\right)>0\\ D^{-}\geq 0,D^{g}\geq 0,D^{b}\geq 0 \end{array}$$

where, $D^{-}$, $D^{g}$ and $D^{b}$ represent the slack variable of input, desirable output and undesirable output, respectively. λ symbolizes the weighting vector. $\rho^{*}$ is a coefficient related to CLGUE.

#### 3.1.2. Mediating Effect Model

On a basis of previous analysis, CLT could prompt CLGUE through the mediator of management scale. Referring to the mediation effect analysis framework proposed by Baron and Kenny (1986) [58], combined with the research hypothesis of this paper, the following hierarchical regression model was constructed:

(3) $$CLGUE_{i,t}=\alpha_{0}+\alpha_{1}CLT_{i,t}+\alpha_{2}Control_{i,t}+\varepsilon_{i,t}$$

(4) $$CLMS_{i,t}=\beta_{0}+\beta_{1}CLT_{i,t}+\beta_{2}Control_{i,t}+\varepsilon_{i,t}$$

(5) $$CLGUE_{i,t}=\gamma_{0}+\gamma_{1}CLT_{i,t}+\gamma_{2}CLMS_{i,t}+\gamma_{3}Control_{i,t}+\varepsilon_{i,t}$$

where i represents the region; t signifies the year; $CLGUE_{i,t}$ is CLGUE for region i within time t; $CLT_{i,t}$ means the proportion of CLT in the total cultivated area for region i within time t; $CLMS_{i,t}$ means the rural per capita sown area for region i within time t; $Control_{i,t}$ represents the control variables, including natural conditions (MCI), the level of regional science and technology (RST), financial expenditure on agriculture (FEA), the level of industrialization (IL), and geographical conditions (GCR), and $\varepsilon_{i,t}$ is the error term. 

According to Baron and Kenny (1986) [58], the mediation effect is supported if the following preconditions can be achieved: Firstly, the regression coefficient $\alpha_{1}$ in Formula (3) should be significant, which indicates that the explanatory variable is significantly related to the explained variable. Secondly, the regression coefficient $\beta_{1}$ in Formula (4) should be significant, which indicates explanatory variable is significantly related to mediating variable. Thirdly, the regression coefficient $\gamma_{2}$ in Formula (5) should be significant, and simultaneously $\gamma_{1}$ in Formula (5) needs to be NOT significant (complete mediating effect), or $\gamma_{1}$ in Formula (5) is significant and the regression coefficient value needs to be less than $\alpha_{1}$ in Formula (3) (partial mediating effect). 

#### 3.1.3. Threshold Regression Model

As mentioned above, the relationship between CLT and CLGUE might be non-linear. Thus, panel threshold modeling proposed by Hansen (1999) [59] is further utilized in the analysis. Specifically, this research selected CLT as the threshold variable to investigate the non-linear affiliation between CLT and CLGUE. The model is established as follows:

(6) $$\begin{array}{c} CLGUE_{i,t}=\delta_{0}+\delta_{1}CLT_{i,t}I\left(CLT\leq \varphi_{1}\right)+\delta_{2}CLT_{i,t}I\left(\varphi_{1}<CLT\leq \varphi_{2}\right)+\cdots \\ +\delta_{n}CLT_{i,t}I(\varphi_{n-1}<CLT\leq \varphi_{n})+\delta_{n+1}CLT_{i,t}I(CLT>\varphi_{n})\\ +\delta_{\omega }Control_{i,t}+\varepsilon_{i,t} \end{array}$$

where I(·) is an indicator function, $\varphi_{1}$ $\varphi_{2}$ $\varphi_{3}$… $\varphi_{n}$ are the specific threshold values. The threshold regression test in Formula (6) consists of two components: First, it is necessary to verify whether there is a threshold effect, and the numbers of thresholds. Second, the paper will check the theoretical asymptotic distribution, establish confidence intervals for parameters to be estimated, and use the bootstrap analysis to evaluate the statistical significance of the threshold. 

### 3.2. Variable Selection and Data Description

(1) Explained Variables. For CLGUE, the super-efficient SBM model was used to evaluate its index. In view of CLGUE, the availability of research data and the relevant literature [9,16,25], twelve variables were selected in our work to construct the evaluation criteria for CLGUE, involving three categories of input indicators (i.e., desirable and undesirable output indicators) (Table 1).

Chemical fertilizers, pesticides, agricultural films, agricultural diesel fuel, agricultural irrigation, agricultural farming and agricultural machinery were regarded as the carbon emission sources for the use of cultivated land in this work. Multiplying the above indicators by the related carbon emission coefficient, the total carbon emission of cultivated land use was gained. It is calculated by the following formula [6]:

(7) $${CECLU}_{i}=\sum C_{i}=\sum T_{i}\cdot \delta_{i}$$

where, $CECLU_{i}$ is the carbon emission for all varieties of carbon sources of cultivated land utilization; $T_{i}$ means the amount of the i-th carbon source; $\delta_{i}$ represents the coefficient of the i-th carbon source. It is known from references [6,60,61] the carbon source and coefficient involve chemical fertilizer (0.895 6, kg C/kg), pesticide (4.394 1, kg C/kg), agricultural film (5.180, kg C/kg), agricultural machinery gross power (312.6 kg, C/kW), agricultural irrigation (5, kg/hm^2^), agricultural cultivation (312.6, kg C/km^2^) and agricultural machinery (25 kg C/hm^2^).

The pollution of cultivated land use is generally non-point source pollution in the process of cultivated land use, which refers to the environmental pollution from pollutants by land runoff and subsurface filtration, with the features of dispersion and concealment [14]. It is reflected in nitrogen and phosphorous losses in fertilizers (10,000 tons), pesticide losses (10,000 tons) and agricultural plastic film residues (10,000 tons). According to reference [9,25], losses of nitrogen (phosphorous) fertilizers, pesticides and agricultural film were utilized to indicate the pollutant emissions from cultivated land use. Based on the manual of agricultural pollution source coefficients published by the National Pollution Source Survey, and considering regional differences, the relevant loss coefficient was evaluated.

(2) Explanatory Variables. The core explanatory variable was CLT, expressed as the area proportion of CLT to household contracted cultivated land under the HRS.
(3) Mediating Variables. The purpose of the CLT was to complete the large-scale management of cultivated land [38]. The CLMS can directly represent the purpose, and the present study selected the per capita sown area to represent CLMS in accordance with the work by Ma et al. (2019) [26].
(4) Control Variables. The control variables were put forward in order to more accurately measure the effect of CLT on CLGUE. Combining the existing relevant studies [25,26], the control variables of the present paper were selected as following: natural conditions (MCI), which is represented by multiple crop index; the level of regional science and technology (RST), which is represented by the ratio of financial expenditure on science and technology; financial expenditure for agriculture (FEA), which is represented by the proportion of financial expenditure for agriculture; the level of industrialization (IL), expressed as the ratio of industrial added value to GDP; geographical conditions (GCR), which is represented by the ratio of affected area of crops in the total sown area.

Table 2 provides the summary statistics of the variables employed in the empirical analysis. 

### 3.3. Research Region and Data Source

**Figure 3:**
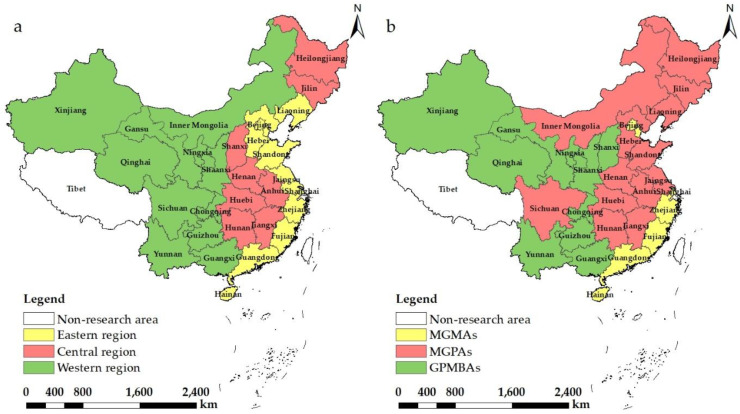
The research area and regional classification. (**a**) three regions in accordance with their locations in eastern, central and western China. (**b**) three food function areas.

China has thirty-four provincial-level administrative institutions, and the level of cropland resources, grain production and agriculture development vary greatly among different regions [14,62]. Given the data availability, Hong Kong, Macao, Taiwan and Tibet were not involved in the empirical research. Thirty provinces of the Chinese mainland were selected as the research objects of this work. It can be known from “National Economic and Social Development Seventh Five–Year Plan” (1985) by CPC Central Committee that these thirty provinces are divided into three regions in accordance with their locations in eastern, central and western China (Figure 3a) [63]. In addition, referring to the Chinese government documents of “Opinions on Reforming and Improving Policies and Measures for Comprehensive Agricultural Development” and “the Outline of the Medium and Long-term Program for National Food Security (2008–2020)”, based on the real situation of grain production and sales volumes in the 30 provinces in recent years, the 30 provinces can be divided into three food function areas: main grain-producing areas (MGPAs), main grain-marketing areas (MGMAs) and grain-producing and marketing balance areas (GPMBAs) (Figure 3b). MGPAs are exclusive economic zones with geographical, soil, climate and technical conditions for planting food crops [38].

The data were collected mostly from the “China Land and Resources Statistical Yearbook”, “China Rural Statistical Yearbook”, “China Environmental Statistical Yearbook”, “China Rural Management Statistical Annual Report” and “China Statistical Yearbook” of the relevant years, as well as from the website of the National Bureau of Statistics of China. The interpolation method was adopted to make up the missing data of the individual years. The data used in our work is the macro-panel data of thirty provinces, which avoids the sample deviation caused by the selection of micro-data [38], so as to better explain the effect of the CLT on CLGUE. 

## 4. Results and Discussion

### 4.1. Measurement and Analysis of CLGUE

**Figure 4:**
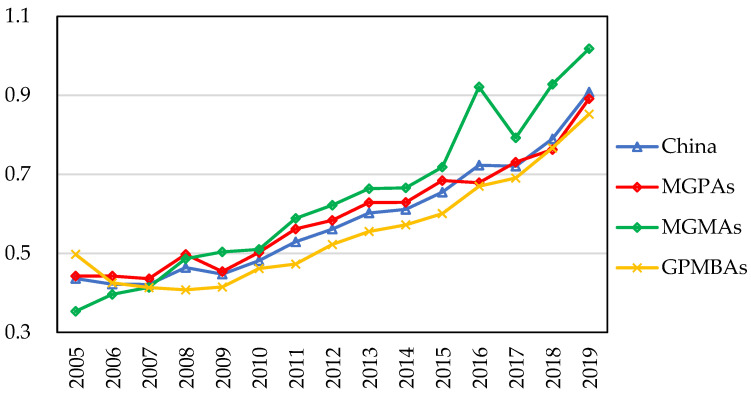
Average value of CLGUE in China, MGPAs, MGMAs, and GPMBAs.

We evaluated the CLGUE of China by Formula (1). Figure 4 shows the overall change of the average value of CLGUE in China and the functional region of grain production from 2005 to 2019. The CLGUE as a whole was on the rise, from 0.440 in 2005 to 0.913 in 2019, with the average annual increase rate of 5.47%. Furthermore, CLGUE was on the rise in all grain functional areas, but the average annual growth rate was significantly different. The average annual growth rate was 5.35, 8.28, and 4.17 in the MGPAs, MGMAs and GPMBAs, respectively. This changing trend is attributable to agricultural science and technology advancements [64], cultivated land fallow [65], the investment in high standard cultivated land, as well as the policy introductions or amendments promulgated by the Chinese government, including “Environmental Protection Law”, “Solid Waste Pollution Prevention Law”, “Soil and Water Conservation Law” and “Water Pollution Control Action plan” [21]. 

**Figure 5:**
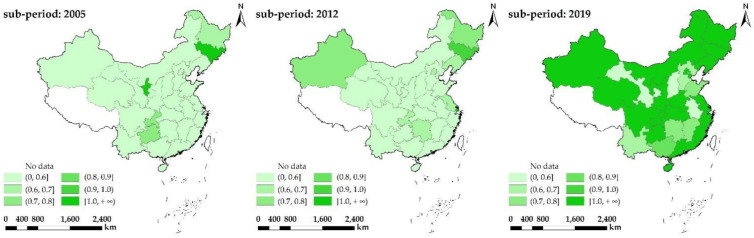
The spatial-temporal evolution of CLGUE.

In order to better visualize the spatial-temporal evolution of CLGUE, the corresponding geographic distribution of CLGUE is shown in Figure 5. According to reference [66], the regions with the efficiency of [1, +∞), (0.9, 1), (0.8, 0.9], (0.7, 0.8], (0.6, 0.7] and (0, 0.6] could be divided into the efficient, high-efficiency, relatively high-efficiency, medium-efficiency, relatively low-efficiency and low-efficiency groups, respectively. 

In 2005, only Jilin and Ningxia fell into the efficient groups, while Heilongjiang and Chongqing belonged to the relatively high-efficiency or medium-efficiency groups. By contrast, 24 provinces belonged to the low-efficiency groups, and their GUECL values were lower than 0.6. In 2012, Jilin and Ningxia were removed from the list of the efficient groups, while Shanghai was moved into the efficient groups. The spatial scope of the relatively high-efficiency and medium-efficiency groups was extended to the original provinces. Beijing, Liaoning, Jiangsu, Xinjiang, Chongqing and Hunan were taken out from the low-efficiency groups. By the contrary, the rest provinces still remained at the low-efficiency group level. In 2019, with a further expansion in the spatial scope of the efficient groups, there was a large increase in the number of relevant provinces, of which Hebei, Jiangxi, Shandong, Hunan, Guangxi, Yunnan and Gansu reached the relatively high-efficiency groups, the medium-efficiency groups or the relatively low-efficiency groups. With the number of relevant provinces in decline continuously, the spatial scope of low-efficiency groups showed a trend of significant shrinkage. Only Shanxi, Anhui, Yuan and Gansu remained within the low-efficiency groups. On the whole, the CLGUE exhibited an obvious heterogeny across all provinces in China. 

Over time, more and more provinces have high CLGUE values. The efficient groups have expanded in general terms and the low-efficiency groups have generally shrunk. China has successfully promoted the low-carbon and ecological growth of agricultural economics and improved the utilization efficiency of cultivated land [67]. In the context of ecological environment friendly and resources sustainable use, the cultivated land use in China has changed to be green and efficient [23].

### 4.2. The Results of Correlation Analysis and Linear Fitting

Table 3 illustrates the results of the Pearson pairwise product–moment correlation matrix between variables. The results demonstrated that there were significant correlations among the major variables (Pearson coefficients ranging from 0.093 to 0.613). Besides, the sample data was free from multi-collinearity (all Pearson coefficients less than 0.7), a situation which is a fundamental prerequisite of regression analysis [68].

**Figure 6:**
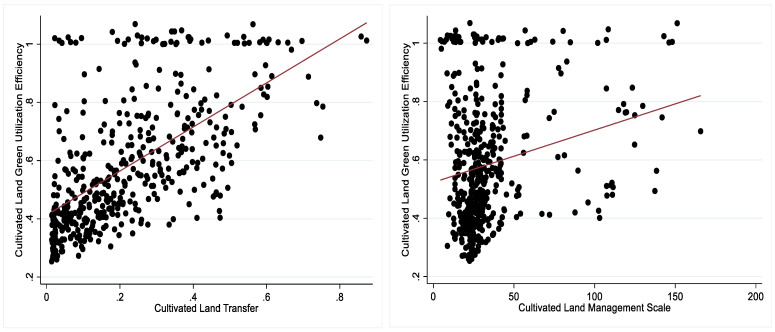
Linear Fitting Diagram. Note: The red line is a linear trend line fitted from all the scatter points.

In order to preliminary analyze the effects of CLT and CLMS on CLGUE, the paper used a linear fitting diagram to display the relationships among the three major variables (Figure 6). As shown in the linear fitting diagram, the relationship between CLT and CLGUE was positive, the same as the relationship between CLMS and CLGUE.. Hypothesis 1 was preliminarily verified. Ensuring the rigor and accuracy, econometric models needed to be tested further in the following research.

### 4.3. Mediating Effect Regression Results

According to the test Formulas (3)–(5) of the mediation effect constructed above, further empirical analysis was performed on the influence mechanism of CLT and CLMS on CLGUE. The data are presented in Table 4. In Model 1, CLT was positively related to CLGUE with the regression coefficient of 0.6361 (*p* < 0.01), indicating that hypothesis 1 was supported. In Model 2, CLT was positively related to CLMS with the regression coefficient of 0.6504 (*p* < 0.01); thereby, hypothesis 2 was verified. Further, in Model 3, when CLT and CLMS were added in Equation (3) simultaneously, it demonstrated that the regression coefficient of CLMS on CLGUE was 0.1115 (*p* < 0.01) and the regression coefficient of CLT on CLGUE was 0.5636 (less than 0.6361) (*p* < 0.01). Therefore, CLT could enhance CLGUE through the partial mediating effect of CLMS. Hypothesis 3 and 4 were supported.

### 4.4. Threshold Effect Regression Results

Referring to the test Formula (6) of the threshold effect constructed, the paper explored whether there were differences in how CLT promoted CLGUE under different levels of CLT. The results are shown in Table 5. Accordingly, the single threshold successfully completed the F-test, but both the double and the triple threshold failed the F-test. It could be considered that the model had a single threshold with a threshold value of 0.3552. Therefore, in order to analyze the correlation between variables, a single threshold pattern needs to be established. The results are presented in Table 6.

As illustrated in Table 6, when CLT was not higher than 0.3552, for each additional unit of CLT, CLGUE could increase by 0.7645 units (*p* < 0.01). Nevertheless, when the level of CLT increased and the regression coefficient was more than 0.3552, for each unit increase in CLT, the CLGUE could increase 0.5296 units (*p* < 0.01). This indicated that CLT could enhance CLGUE comprehensively which was consistent with the results of mediating effect analysis. Besides, when CLT was at a relatively low level, the promotion effect was more obvious. One possible interpretation for this phenomenon is that a large number of rural laborers transferred against the background of the large-scale CLT. The study of Zhang et al. (2017) found that rural labor out-migration leads to high fertilizer use rate [69], which will result in higher carbon emissions from cultivated land utilization. Hou et al. (2021) explored the two-way interaction effect between rural labor transfer and agricultural ecological efficiency. The results showed that there were significantly negative and positive interaction effects between agricultural ecological efficiency and rural labor transfer [70]. As a consequence, the impact of CLT on CLGUE is moderated by other factors, such as the transfer of rural labor.

### 4.5. Heterogeneity Analysis 

The heterogeneity analysis based on different geographical location was used to explore the different effects of CLT on CLGUE. As shown in Table 7, when the sample was divided into eastern, central and western areas based on “National Economic and Social Development Seventh Five–Year Plan (1985)” published by the CPC Central Committee, the CLT was significant at the level of 1%, and the coefficient was positive in all areas, which indicated that as the CLT increases, CLGUE has been significantly improved. In addition, the regression coefficients of CLT on CLGUE in eastern areas were higher than those in western and central areas. This may be attributed to the characteristics of economic reproduction in agriculture. In view of the high level of economic and management systems in the eastern region, and the increasingly active CLT market resulting from the increase in non-agricultural income [54], it is easy to build a regional advantage of CLT on CLGUE.

In order to study the effect of the grain functional areas on CLGUE, in this study, MGPAs, MGMAs and GPMBAs in models 3 were regressed, respectively (Table 7). As shown in Table 6, the CLT was significant at the level of 1%, and the coefficient was positive in all areas, which indicated that as the CLT increases, CLGUE has been significantly improved. In addition, the regression coefficients of CLT on CLGUE in MGPAs were lower than those in MGMAs and GPMBAs. One reason for this phenomenon may be related to the regional differences in ecological efficiency of cultivated land utilization. In other words, medium–high efficiency and high–efficiency provinces are frequently spread in MGMAs and GPMBAs, and low efficiency and medium–low efficiency provinces are concentrated in MGPAs [14]. Furthermore, the provinces in MGPAs show higher carbon emission from the utilization of cultivated land and higher increase rates than other provinces [6], as consumptions and carbon emission in those provinces are higher due to the pressures from agricultural production and grain safety [60]. Above factors resulted in the influence of CLT on CLGUE at a relatively lower level. 

## 5. Conclusions and Policy Recommendations 

### 5.1. Conclusions

Using the super-SBM model, the CLGUE of 30 Chinese provinces from 2005 to 2019 was measured with the carbon emission and non-point source pollution as undesirable outputs. Furthermore, mediating effect model and threshold regression model were employed to investigate the effect of CLT on CLGUE. Based on the empirical results, the major conclusions of this study are given below. 

(1) The CLGUE at the national level as a whole increased persistently, from 0.440 in 2005 to 0.913 in 2019, with the average annual increase rate of 5.47%. Due to the different natural and economic conditions, the CLGUE trends showed significant spatial disparities at both grain functional areas level and provincial level. From a grain functional perspective, CLGUE in the three regions showed an overall upward trend, with the mean yearly growth rate in an order of the MGMAs > MGPAs > GPMBAs. From the provincial level, more and more provinces have high CLGUE values over time. The efficient groups expanded in general terms and the low-efficiency groups generally shrank.

(2) The benchmark regression results indicated that CLT could promote CLGUE directly, besides the results passed the robustness test. The mediation regression results demonstrated that CLT was able to enhance CLGUE indirectly through the mediator of CLMS. The threshold effect test confirmed the existence single threshold, indicating that when the level of CLT was gradually crossing the threshold, the promotion effects of CLT on CLGUE would slow down. Attention should be paid to encouraging the practice of CLT and increasing CLMS of household through policies oriented toward highly ecologically friendly and green-efficient utilization of cultivated land and sustainable regional development.

(3) The heterogeneity analysis indicated that considering geographical location, the promotion effects of CLT on CLGUE in eastern and western areas were relatively larger than those in central area. Besides, the promotion effects of CLT on CLGUE in MGMAs and GPMBAs were much higher than that in MGPAs. For the purpose of enhancing the influence of CLT on CLGUE in central areas and MGPAs, a balance should be achieved in policy making between the pressures from grain safety and ecological protection. 

### 5.2. Policy Recommendation

Based on the empirical findings achieved in this work, several initiatives are proposed. 

Promoting CLT should be listed as the priority direction of cultivated land utilization policy of China. The empirical results demonstrated that CLT could promote CLGUE directly. In the context of HRS and “Three Rights Separation Policy”, the mechanisms of CLT should be made more standard and formal for the purpose of reducing the opportunistic behaviors. Furthermore, stabilizing the expectation of tenure security for farmers who have transferred in cultivated land, and the rental income for farmers who have transferred out cultivated land. The relevant policies should be issued to improve farmers’ willingness of the practice in CLT. In addition, decision makers should also practice management measures such as regulating the behavior of CLT, formulating reasonable CLT pricing models [42], and a variety of operations including family farm, agricultural cooperative, agricultural enterprise management should be encouraged. The empirical results demonstrated that CLT was able to enhance CLGUE indirectly through the mediator of CLMS. Therefore, in order to promote the transformation of cultivated land use to being green and efficient, we should expand the CLMS and strengthen the supporting role of CLMS on CLGUE. Promoting the perfection and maturity of CLT mechanism is a key approach to expand the CLMS [44].

## Figures and Tables

**Table 1:** The statistics for measuring CLGUE.

Primary Indicators	Secondary Indicators	Variables and Descriptions
Inputs	Labor input	AFAHF✕(Total agricultural output/TO) (10^4^ people)
	Land input	Total sown area of crops (10^3^ hectare)
	Capital input	Fertilizer consumption (10^4^ tons)
		Pesticide consumption (10^4^ tons)
		Consumption of agricultural film (10^4^ tons)
		Total agricultural machinery power (10^4^ kw)
		Effective irrigation area (10^3^ hm^2^)
Desirable Outputs	Economic output	Total agricultural output (10^4^ Yuan)
	Social output	Total agricultural output (10^4^ tons)
	Environmental output	The total carbon sink (10^4^ tons)
Undesirable Outputs	Pollution emission	The total loss of fertilizer nitrogen (phosphorous), pesticides and agricultural film (10^4^ tons)
	Carbon emission	The carbon emissions from cultivated land utilization (10^4^ tons)

Note: AFAHF represents the abbreviation of agricultural, forestry, animal husbandry and fishery practitioners; TO represents the abbreviation of total output values of agriculture, forestry, animal husbandry and fishery.

**Table 2:** Descriptive Statistics of Variables.

Variables	Mean	Std. Dev.	Minimum	Maximum
Cultivated land green utilization efficiency (CLGUE)	0.585	0.216	0.254	1.069
Cultivated land transfer (CLT)	0.227	0.175	0.0136	0.873
Cultivated land management scale (CLMS)	0.346	0.269	0.0437	1.657
Regional natural conditions (MCI)	128.3	35.66	41.46	221.7
The level of regional science and technology (RST)	1.807	1.416	0.223	7.202
Financial expenditure for agriculture (FEA)	10.47	3.338	2.133	18.97
The level of industrialization (IL)	45.54	8.472	16.16	61.50
Regional geographical conditions (GCR)	32.01	24.61	0	174.3

**Table 3:** Correlation Analysis of Variables.

	CLGUE	CLT	CLMS	MCI	RST	FEA	IL	GCR
CLGUE	1							
CLT	0.613 ***	1						
CLMS	0.223 ***	0.093 **	1					
MCI	−0.0670	0.160 ***	−0.322 ***	1				
RST	0.346 ***	0.654 ***	−0.244 ***	0.185 ***	1			
FEA	0.085 *	−0.094 **	0.395 ***	−0.307 ***	−0.434 ***	1		
IL	−0.390 ***	−0.350 ***	0.0540	0.150 ***	−0.271 ***	−0.121 ***	1	
GCR	−0.405 ***	−0.457 ***	−0.0470	−0.163 ***	−0.304 ***	0.117 **	0.082 *	1

Note: * *p* < 0.1, ** *p* < 0.05, *** *p* < 0.01.

**Table 4:** Hierarchical Regression Results.

Variables	Model (1)	Model (2)	Model (3)
	CLGUE	CLMS	CLGUE
CLT	0.6361 ***	0.6504 ***	0.5636 ***
	(10.0718)	(5.4464)	(8.1720)
CLMS			0.1115 ***
			(3.0206)
MCI	−0.0007 ***	−0.0022 ***	−0.0005 **
	(−3.5498)	(−7.3549)	(−2.4322)
RST	−0.0063	−0.0627 ***	0.0007
	(−0.6884)	(−5.1407)	(0.0691)
FEA	0.0051 *	0.0185 ***	0.0031
	(1.8402)	(5.2587)	(1.0509)
IL	−0.0045 ***	0.0060 ***	−0.0052 ***
	(−3.9942)	(4.1403)	(−4.4843)
GCR	−0.0017 ***	−0.0005	−0.0017 ***
	(−3.5096)	(−1.0507)	(−3.4155)
cons	0.7535 ***	0.1449 *	0.7374 ***
	(9.5532)	(1.7231)	(9.3637)
*N*	450	450	450
adj. *R*^2^	0.458	0.308	0.470
F	F (6,443) = 81.82	F (6,443) = 18.29	F (7,442) = 68.87

Note: *t* statistics in parentheses; * *p* < 0.1, ** *p* < 0.05, *** *p* < 0.01.

**Table 5:** Thresholds Corresponding to Different CLT Levels.

Model	F-Value	*p*-Value	Critical Value			Threshold Value	95% Confidence Interval	
			10%	5%	1%			
Single threshold	20.40 *	0.093	19.6500	23.6583	32.0941	0.3552	0.3493	0.3565
Double threshold	4.83	0.827	15.7092	17.9890	24.9962			
Triple threshold	10.90	0.473	18.8730	24.3689	30.2588			

Note: * *p* < 0.1.

**Table 6:** Threshold Regression Results.

Variables	Regression Coefficients	Standard Error	T-Value	*p*-Value	95% Confidence Interval	
CLGUE·I (CLMS ≤ 0.3552)	0.7645 ***	0.1334	5.73	0.000	0.4916	1.0373
CLGUE·I (CLMS > 0.3552)	0.5296 ***	0.0849	6.24	0.000	0.3561	0.7032
MCI	−0.0009	0.0010	0.97	0.340	−0.0029	0.0010
RST	0.0359 ***	0.0120	2.99	0.006	0.0113	0.0604
FEA	−0.0066	0.0054	1.23	0.228	−0.0176	0.0044
IL	−0.0107 ***	0.0022	4.79	0.000	−0.0153	−0.0062
GCR	−0.0013 ***	0.0004	3.28	0.003	−0.0021	−0.0005
cons	1.0930 ***	0.1789	6.11	0.000	0.7271	1.4588

Note: *** *p* < 0.01.

**Table 7:** Heterogeneity analysis of effects of CLT on CLGUE.

Variables	Division by Geographical Location			Division by Grain Functional		
	Eastern Areas	Central Areas	Western Areas	Main Grain -Producing Areas	Main Grain -Marketing Areas	Grain-Producing & Marketing Balance Areas
	CLGUE	CLGUE	CLGUE	CLGUE	CLGUE	CLGUE
CLT	0.6699 ***	0.5860 ***	0.6675 ***	0.4382 ***	0.6783 ***	0.6481 ***
	(7.9870)	(3.0154)	(5.2826)	(3.5554)	(6.1136)	(4.3950)
MCI	−0.0005	−0.0016 ***	0.0007	−0.0020 ***	0.0007	0.0018 **
	(−1.1732)	(−4.7078)	(1.0382)	(-7.8225)	(1.3163)	(2.3415)
RST	0.0020	−0.0468 **	0.1208 ***	−0.0011	0.0034	0.0914 ***
	(0.1915)	(−2.1303)	(3.7356)	(−0.0781)	(0.2513)	(2.6983)
FEA	0.0132 ***	0.0005	0.0038	−0.0018	0.0192 ***	0.0093
	(2.9591)	(0.0633)	(0.6671)	(−0.3621)	(3.0400)	(1.5791)
IL	−0.0040 ***	−0.0056 **	−0.0024	−0.0097 ***	−0.0045 ***	−0.0014
	(−3.0460)	(−1.9957)	(−1.1158)	(−4.6191)	(−2.6961)	(−0.5800)
GCR	−0.0007	−0.0020 **	−0.0017 ***	−0.0024 ***	−0.0009	−0.0016 **
	(−1.5274)	(−2.2258)	(−2.8124)	(−3.9650)	(−1.5338)	(−2.5020)
cons	0.5711 ***	1.0670 ***	0.3730 **	1.3277 ***	0.3806 ***	0.1552
	(5.9318)	(5.7331)	(2.0906)	(10.4772)	(2.9352)	(0.8061)
*N*	165	120	165	195	105	150
adj. *R*^2^	0.554	0.460	0.475	0.570	0.513	0.454
F	34.99	17.91	25.72	43.92	19.27	21.66

Notes: 1. ** *p* < 0.05, *** *p* < 0.01; 2. Eastern areas: Beijing, Tianjin, Hebei, Liaoning, Shanghai, Jiangsu, Zhejiang, Fujian, Shandong, Guangdong, Hainan; Central areas: Shanxi, Jilin, Heilongjiang, Anhui, Jiangxi, Henan, Hubei, Hunan; Western areas: Inner Mongolia, Guangxi, Chongqing, Sichuan, Guizhou, Yunnan, Shaanxi, Gansu, Qinghai, Ningxia, Xinjiang. 3. Main grain production areas (MGPAs): Hebei, Inner Mongolia, Liaoning, Jilin, Heilongjiang, Jiangsu, Anhui, Jiangxi, Shandong, Henan, Hubei, Hunan, Sichuan; Main grain-marketing areas (MGMAs): Beijing, Tianjin, Shanghai, Zhejiang, Fujian, Guangdong, Hainan; Grain-producing & marketing balance areas (GPMBAs): Shanxi, Ningxia, Qinghai, Gansu, Yunnan, Guizhou, Chongqing, Guangxi, Shaanxi, Xinjiang.

## Data Availability

The data will be made available to the reader upon request.
